# The pharmaceutical practice of mask distribution by pharmacists in Taiwan’s community pharmacies under the Mask Real-Name System, in response to the COVID-19 outbreak

**DOI:** 10.1186/s12962-020-00239-3

**Published:** 2020-10-19

**Authors:** Che-huei Lin, Ya-Wen Lin, Jong-yi Wang, Ming-hung Lin

**Affiliations:** 1 Department of Pharmacy and Master Program, Tajen University, Pintung, 90741 Taiwan; 2 Department of College Public Health, China Medical University, Taichung, 40402 Taiwan; 3 Department of Health Services Administration, China Medical University, Taichung, 40402 Taiwan; 4 Fulun Chain Drugstore, Nantou, 54062 Taiwan; 5 Department of College Business Administration, National Chiayi University, Chiayi, 60054 Taiwan; 6 School of Nursing, China Medical University, Taichung, 40402 Taiwan

**Keywords:** Community pharmacies, Mask, COVID-19

## Abstract

**Background:**

Pharmacists hold to their promise to foster, implement and promote the health of the population and to prevent disease, given their knowledge, skills, and proximity to the locals. The objective of this study was to foster equality and cost-effectiveness in the distribution and sale of masks to all Taiwanese citizens, in response to the COVID-19 pandemic.

**Methods:**

All 6336 special community pharmacies participating in the NHI (National Health Insurance) served as mask-selling sites. Access to masks by citizens was determined and controlled, based on the weekly rationing of the number of purchasable masks per citizen and the last digit of their NHI card number. Masks were available on different weekdays for holders of cards ending with odd and even numbers, except on Sundays, when everyone was eligible to buy a mask.

**Results:**

Implementing the program has provided equal access to masks for all citizens across Taiwan. It has stabilized the pricing of masks and mitigated the public’s anxiety of a perceived likely market shortage.

**Conclusion:**

The community pharmacy-based approach to the distribution of prevention face masks to citizens represents a new and innovative engagement of pharmacists in public health promotion and protection initiatives. Community pharmacies can greatly improve the efficiency, reliability, and cost-saving of the distribution of public health resources to local communities, especially in the face of an epidemic.

## Introduction

At the core of founding an informed public health management strategy, there should be an emphasis on the need to engage and empower individuals and communities to assure their own health, and that of others, while mitigating and responding to the public health risks and related exposure within the population [1].

This is a defining component of the typical health services utilization process and the behavior of all members of society, despite their entry point into the health system [2]. The community pharmacy-based program for the ‘Mask Real-Name System’ demonstrated the potential competitive value that is inherent in pharmacists who enhance the distribution-related outcomes of scarce public health management resources to target populations and communities. Specifically, the active involvement of pharmacists across Taiwan in the implementation of public health promotion and disease control efforts is associated with optimizing the outcomes, by enhancing the management of such efforts for the cost-effectiveness and reliability of their reach and impact on society [3, 4].

The prospects for realizing and sustaining a healthy population cannot be assumed where there is no system-wide commitment to embrace and promote well-informed, value-laden public health and protection strategies by all the relevant stakeholders in public health. At the core of the fundamental features of an informed public health management strategy, there should be an emphasis on the need to engage and empower individuals and communities to assure their own health and that of others, while mitigating and responding to the public health risks and related exposure in the population [1]. National public health efforts, especially in the wake of a pandemic, can be overwhelming to health systems, in terms of the costs and implementation technicalities, among other challenges. To this end, the critical value of investing in innovative population health management strategies, as a safeguard for assuring the sustainable competitive performance of the health system of a country, cannot be over-emphasized.

Pharmacists boast that they have the knowledge, skills, professional expertise and proximity advantage that are needed to facilitate and contribute value-laden outcomes related to the implementation of the health promotion and disease prevention efforts by the government [1]. They are a defining component of the typical health services utilization process and behavior by all members of the society, despite their entry point into the health system [2]. In Taiwan, community pharmacies have proved to be an invaluable resource for the government in helping to implement various health promotion initiatives. From their active involvement in the recovery of residual medicines, discarded medicines, and AIDS syringe recycling, to anti-drug education and campaigns in primary schools and community drug safety workshops, as well as smoking cessation and advocating the preventation of the betel nut hazard, community pharmacies play an important role in the promotion of public health prevention and treatment in Taiwan (Additional file 1).

During the continuous spread of the novel coronavirus epidemic, which started in February 2020, and its related effect on fueling the global phenomenon of chaotic mask purchases, the Taiwanese people were seen lining up overnight to buy masks, which caused panic and a crisis throughout the country. The Taiwan government quickly announced a ban on the export of masks (2020 Feb 6) and the national mask factories were requisitioned to distribute them uniformly [5]. At the same time, the government announced the implementation of the “Mask Real-Name system” policy for face mask distribution to individuals and families across the nation (Additional file 2). This policy was the outcome of a high-profile meeting of government officials from the Epidemic Prevention Bureau and the Center for Disease Control (CDC) of Taiwan, experts from the Taiwan Pharmacists Association, as well as representatives from the Chunghwa Post, concerning the feasibility of the “Mask Real-Name system “amidst the fight against the ongoing COVID-19 pandemic. By March (1 month after the implementation), the Federation of Taiwan Pharmacists Associations, based on the sale of masks in pharmacies in all the counties and cities, found that adults currently use three, and children use five, masks per week, and each pharmacy receives 250 customers per day. In fact, the national mask coverage rate is merely 40%, on average (Statistics of the Federation of Taiwan Pharmacists Associations, March 2020). Since April 17, when the ‘Mask Real-Name system’ was put into operation, 6336 community pharmacies across Taiwan have seen a total of 9,750,000 customers buying masks under the Real-Name system per week, and a total of 39,000,000 customers (250 customers per day) per month. In addition, according to statistics from the National Health Insurance Administration of the Ministry of Health and Welfare, 20.41 million people have bought masks since the Mask Real-Name System began on February 6. As of April 29, 20% (about 3.99 million people) have purchased masks from the Internet and physical stores, 7% (about 1.45 million people) have only used the Internet (including supermarkets) to buy masks, and 73% (about 14.96 million people) have bought masks from physical channels, such as community pharmacies or health clinics (Source: Ministry of Health and Welfare).

Given their proven performance record as being a reliable resource for helping to implement public health promotion initiatives by the Taiwanese government, community pharmacies were enlisted to assist in the distribution of face masks to citizens across the country. The objectives were to foster the efficiency, reliability, and cost-effectiveness in the distribution process and to enhance the equality and fairness of access by all Taiwanese citizens to face masks.

## Program description

The Taiwanese government is on record as having the highest coverage rate (99%) for National Health Insurance (NHI) and the most comprehensive health insurance database in the world. In total, the 6336 special community pharmacies participating in the NHI were used as mask-selling sites in the program. In particular, all face masks that were available to the general public in Taiwan were distributed by pharmacists in community pharmacies. The access to masks was determined and controlled, based on the value of the last digit of the NHI card number of individual citizens. More specifically, for all citizens with NHI cards ending with an odd number, masks were only available on Mondays, Wednesdays, and Fridays, while for those ending with an even number; masks were available on Tuesdays, Thursdays, and Saturdays. Face masks were available for purchase on Sundays to all citizens, regardless of the value of the last digit on their NHI (National Health Insurance) card.

According to the announcement of the Ministry of Economic Affairs, the cost of raw materials for masks rose to 118% in April 2020, and climbed to 150% in May. However, the Ministry of Health and Welfare stipulated that the price of masks sold to the public by community pharmacies should be NT$5 per mask (the price of masks before the pandemic was about NT$2 per mask). Besides, all mask factories were dispatched to produce masks. Since February 6, when the Mask Real-Name System was launched, the Central Epidemic Command Center announced further, on July 1, 2020, that the Mask Real-Name System would be extended to December 31, 2020. At the same time, from June 1, the Ministry of Health and Welfare announced that some masks can be freely traded in supermarkets. However, the selling price varied greatly from NT$6 to NT$10 per mask, while the price of masks under the Real-Name System was controlled by the government at NT$5 per mask.

In addition, to mitigate chaotic mask-purchasing behavior and the related risk of prompting undue face mask shortages in the market, as well as the possibility of creating a public panic and a crisis across the nation, the distribution and sale of face masks to citizens via community pharmacies was based on a rationing system [5]. The ration for the number of face masks available for purchase at the start of the program was two face masks per week for each adult and child in the country, but on February 20, the number was increased to four masks per week per individual, for both adults and children. To purchase masks, individuals needed to take their health insurance card to the community pharmacy and swipe it for the purchase, and the data were uploaded to the NHI cloud system to automatically check whether the masks were being bought repeatedly. The system also displayed the available inventory of the purchases. There was also an ongoing effort by the government, in collaboration with Non-Governmental Organizations (NGOs), to develop a “Mask Map” app to provide details concerning the location of nearby pharmacies and the available inventory.

The Ministry of Health and Welfare requires the Department of Social Welfare of local county and city governments to cooperate with the Department of Health and to manage a list for the physically disabled and the elderly who live alone, and the Ministry of Health was required to visit and distribute the masks, together with the social workers. Village officials may carry out the above affairs on behalf of the Ministry of Health.

## Results

The implementation of the program required the collaboration of pharmacists serving within community pharmacies, the finance personnel, information technologists, as well as the producers and distributors of face masks in the nation. The eligible buyers were determined by using the value of the last digit on the NHI card number of each individual and the data of their purchase history from the NHI database. These policy provisions were meant to mitigate the possibility of individuals engaging in multiple mask purchases. Pharmacists in the community pharmacies determined and informed the local residents on the availability of masks and the specific times that they could make their purchases. In making this determination, pharmacists had to consider the available mask inventory levels, relative to the demand for masks by health care personnel within their local communities, as well as the timing when the majority, if not all, of the locals were available to make the purchases.

The involvement of community pharmacists in selling prevention masks greatly enhanced the control of the distribution of face masks in markets across the nation. Notably, this approach to the distribution and sale of masks mitigated the free-market approach to sales, by ensuring that masks were only available for sale to citizens through registered community pharmacies. This eliminated the chaos of having rising market prices, as it offered government authorities the upper-hand in setting and enforcing the mask prices. It was also an effective way of alleviating public anxiety about a likely mask shortage. Above all, it provided a competitive means for safeguarding the healthcare system from undue mask shortages, by prioritizing the demands of the healthcare personnel mask during allocation. Furthermore, details on the travel history of individuals were also filtered through the NHI cloud system, which, in turn, helped to improve the identification of subjects requiring isolation and those violating the isolation mandates of the Taiwanese government in the fight against the COVID-19 pandemic.

## Discussion

The community pharmacy-based approach to the distribution and sale of prevention face masks to citizens for the ongoing COVID-19 pandemic has proven to be a new and innovative engagement of pharmacists in contributing to the efficient, reliable, equitable, and cost-effective implementation of public health promotion and protection initiatives by the government. By providing the increased electronic tracking and reporting of face mask sales in the various communities across the nation, community pharmacies provide the NHI with an informed and reliable mechanism for mitigating chaotic incidents in the distribution, selling, and purchasing of masks across the country [6]. This approach provides a quick way of securing and reporting data to be used for the government’s planning and decisions on the production and distribution of face masks to meet the underlying objective of safeguarding equal access to pandemic prevention resources by all citizens across the nation. In particular, this approach is valuable for enhancing cost-savings by the NHI, as it eliminates the arbitrary setting of mask prices by vendors in the marketplace.

The “Mask Real-Name System 2.0” program is based on the revision of the tax filing software of the Ministry of Finance. It features a stable system and immediate online operation. However, this system needs to be equipped with a card reader to read the health insurance card and it can only operate on a computer, which is inconvenient for mobile phone users and it cannot, therefore, be popularized among the public. To make it accessible for all people, the government immediately introduced the mobile phone APP, in order to connect with the National Health Insurance system.

To avoid long night-queues to purchase masks, the “Mask Real-Name System 2.0” has changed the “queuing system” into a “registration system”, with the aim of dispersing people, reducing the pressure load on the computer system, and preventing people from queuing up to buy masks.

The Taiwanese government has been engaged in multiple steps from mask exports, to production, pricing to rationing. Such engagement in the Real-Name system has brought many benefits. The price of masks is stable and people can obtain masks at a lower price than in other countries, thus avoiding the epidemic prevention gap caused by the inequality between the wealthy and the poor. In addition, the government’s capital and manpower, such as the army’s support for mask production, have also been key in increasing the mask production capacity. Therefore, the implementation of the Real-Name system allows people to acquire a certain number of masks on a regular basis, which effectively reduces the crowds.

Some of the shortcomings and difficulties faced by the selling of Real-Name masks in community pharmacies are as follows: (1) Community pharmacies need to pack and sell the masks. Each pharmacy needs to handle an average of 2000 masks per day. In addition to the daily pharmacy sales, the community pharmacies are required to employ more people; (2) In some community pharmacies, one person is responsible for the whole pharmacy business, and some pharmacists are either in a poor physical condition, pregnant, or unable to provide services for long periods of time, due to family factors, or age; (3) the Taiwanese government has extended the Mask Real-Name System till the end of December 2020. Pharmacists in community pharmacies are generally exhausted, both physically and mentally, because of irrational people who come to buy masks, so they often suffer abuse and their personal safety is threatened.

However, the planned economical mode of the government will definitely stifle the mechanisms and advantages of the free market. Under the government pricing control, people can obtain masks at cheaper prices. In addition, a fixed quota seems to be equal for everyone, and it does not reflect the differences in personal and family needs. For example, medical personnel or business sales personnel actually do need a different number of masks every week. It is not feasible to adjust the rations, so as to quantify the differences in lifestyles. Moreover, it may also lead to ethnic opposition, occupational discrimination, and other related issues.

Furthermore, given that the program is integrated with the NHI database, and hence, the electronic health and medical records of patients across the nation, it allows pharmacists in community pharmacies to assist in the tracking and filling of signed prescriptions for local patients with chronic diseases. In particular, it promises to improve the coordination of supplies for critical medicines and related pharmaceuticals to community pharmacies, which is critical for reducing the number of people going to a hospital for non-critical or avoidable healthcare concerns. Moreover, given their professional knowledge and skills, pharmacists serving in community pharmacies can also provide professional drug consultation services to locals [2]. All of this has the ultimate value of helping to reduce the number of people having to visit hospitals across the country, which, in turn, reduces the undue spread of COVID-19, by mitigating unnecessary travel and overcrowding in hospitals. This is a critical measure for safeguarding the sustainability of the national health system in the successful fight against pandemics.

## Conclusions

As Taiwan is fighting ‘COVID-19’, we use the advantages of a complete NHI information system and unite it with community pharmacists, in order to penetrate each community. The pharmaceutical practice of community pharmacists distributing masks to Real-Name buyers, in response to the COVID-19 pandemic, is an innovative public health management program that optimizes the value-based exploitation of pharmacists, community pharmacy networks (Taiwan telcom VPN), and available technology to allow for an efficient, cost-effective, reliable, and equitable population reach. This program, therefore, enables pharmacists to impact the effective management of population health by collaborating with the government and companies to ensure that there are sufficient mask supplies and well-coordinated sales to citizens across the country. Given the technology-centric nature of the program, it provides assurance to pharmacists engaging in population health management initiatives of a competitive return on value (Additional files 1 and 2).

## **Supplementary information**

**Additional file 1.** Taiwanese citizens buy mask in queue outside the pharmacy.

**Additional file 2.** Taiwanese citizens line up outside the pharmacy to buy masks.

## Data Availability

Not applicable.
