# Supplementary figures and images for: The pharmaceutical practice of mask distribution by pharmacists in Taiwan’s community pharmacies under the Mask Real-Name System, in response to the COVID-19 outbreak
Source: Cost Eff Resour Alloc. 2020 Oct 19;18:45. doi: 10.1186/s12962-020-00239-3 (PMC7570415; doi:10.1186/s12962-020-00239-3)

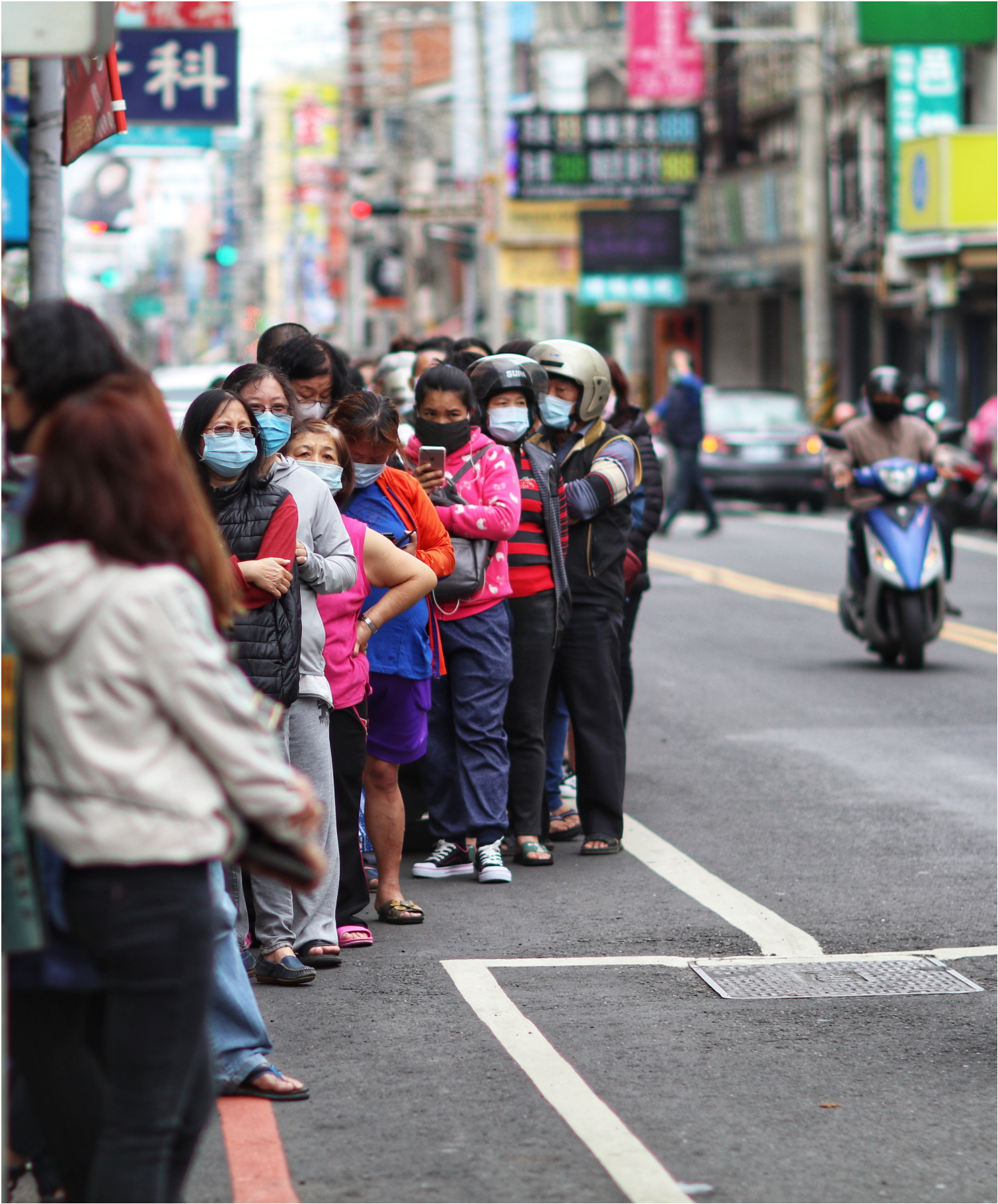

Supplement: Supplementary file 1 — Additional file 1. Taiwanese citizens buy mask in queue outside the pharmacy. [file 12962_2020_239_MOESM1_ESM.pdf]

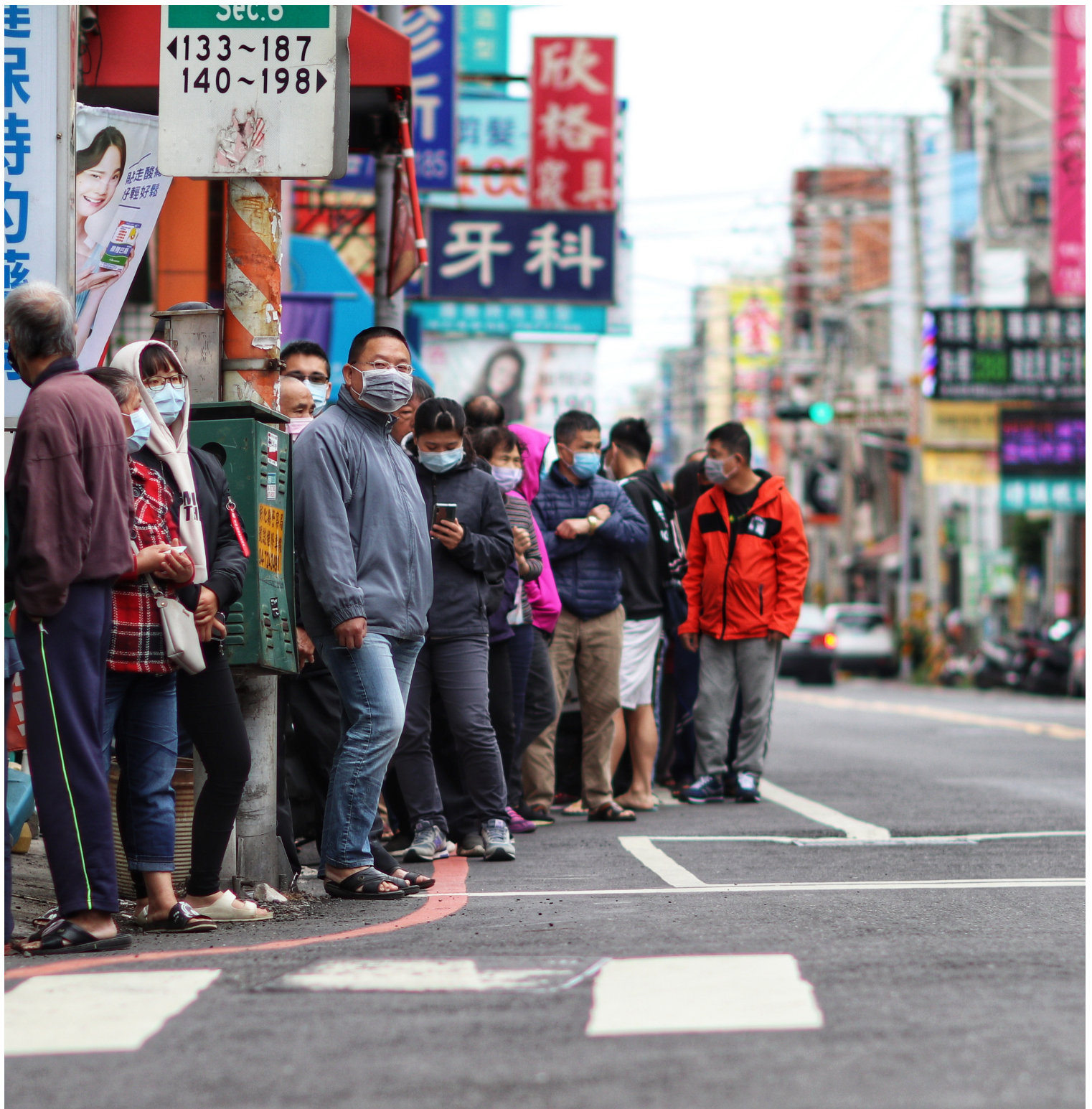

Supplement: Supplementary file 2 — Additional file 2. Taiwanese citizens line up outside the pharmacy to buy masks. [file 12962_2020_239_MOESM2_ESM.pdf]
